# Time-Distributed Framework for 3D Reconstruction Integrating Fringe Projection with Deep Learning

**DOI:** 10.3390/s23167284

**Published:** 2023-08-20

**Authors:** Andrew-Hieu Nguyen, Zhaoyang Wang

**Affiliations:** 1 Neuroimaging Research Branch, National Institute on Drug Abuse, National Institutes of Health, Baltimore, MD 21224, USA; hieu.nguyen@nih.gov; 2 Department of Mechanical Engineering, The Catholic University of America, Washington, DC 20064, USA

**Keywords:** three-dimensional image acquisition, three-dimensional sensing, single-shot imaging, fringe-to-phase transformation, convolutional neural network, deep learning

## Abstract

In recent years, integrating structured light with deep learning has gained considerable attention in three-dimensional (3D) shape reconstruction due to its high precision and suitability for dynamic applications. While previous techniques primarily focus on processing in the spatial domain, this paper proposes a novel time-distributed approach for temporal structured-light 3D shape reconstruction using deep learning. The proposed approach utilizes an autoencoder network and time-distributed wrapper to convert multiple temporal fringe patterns into their corresponding numerators and denominators of the arctangent functions. Fringe projection profilometry (FPP), a well-known temporal structured-light technique, is employed to prepare high-quality ground truth and depict the 3D reconstruction process. Our experimental findings show that the time-distributed 3D reconstruction technique achieves comparable outcomes with the dual-frequency dataset (p = 0.014) and higher accuracy than the triple-frequency dataset (p = 1.029 × $10^{-9}$), according to non-parametric statistical tests. Moreover, the proposed approach’s straightforward implementation of a single training network for multiple converters makes it more practical for scientific research and industrial applications.

## 1. Introduction

Three-dimensional (3D) reconstruction, a subfield within computer vision, has gained exceptional popularity as a measurement tool in recent decades, owing to its inherent advantages in capturing real-world objects’ visual appearance and geometric shape. The process of 3D reconstruction involves using computer vision algorithms and image processing techniques to analyze a set of representative two-dimensional (2D) images and generate a 3D digital point-cloud model of an object or scene. The demand for 3D shape reconstruction is evident across many applications in various fields such as vision-guided robots, visual inspection, face recognition, autonomous navigation, medical imaging, driverless vehicles, 3D entertainment, archaeology, and gaming [1,2,3,4,5].

**Figure 1:**
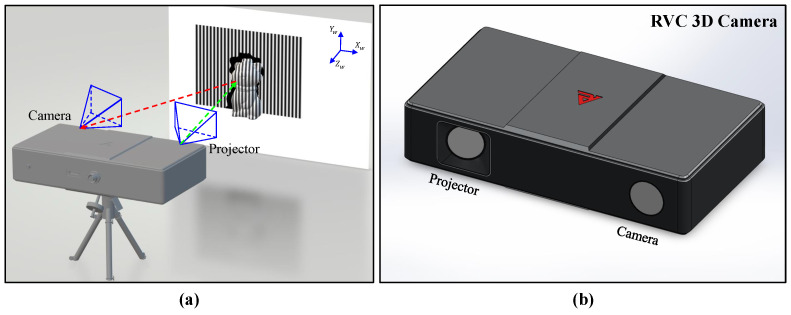
(**a**) Illustration of a 3D reconstruction system and process; (**b**) an RVBUST RVC 3D Camera employed in this work.

There are two primary categories of 3D shape reconstruction techniques: active methods and passive methods. Typical active methods encompass time-of-flight, structured light, optical interferometry, laser scanning, computed tomography, etc. [6,7,8,9,10]. On the other hand, popular passive methods comprise stereo vision, photogrammetry, shape from motion, shape from defocus, etc. [11,12,13,14,15]. Active techniques, as opposed to passive ones that solely rely on the natural texture information captured, project known patterns onto the target of interest and observe their deformation, enabling highly accurate depth measurements. Among these active methods, structured-light 3D reconstruction techniques have become increasingly popular in industrial applications due to their extraordinary accuracy and reliability. Figure 1 showcases a typical 3D reconstruction system and the utilization of illuminated patterns for precise 3D reconstruction. A key drawback of such a technique is that its measurement speed is slow when high accuracy is desired since multiple images are required and complicated computations are involved. This well-known limitation has troubled the technical community for many years, until recently when artificial intelligence (AI) provided opportunities to tackle it.

The present world is in an era of big data with tremendous amounts of information and data generated every second, presenting a considerable challenge for relevant personnel in integrating and efficiently utilizing this abundance of data. In recent years, the rise of AI has helped cope with the problem. AI technologies have empowered machines to perform tasks previously considered beyond human capabilities. Deep learning, a collection of learning algorithms and statistical models derived from AI, emulates the human brain’s cognitive processes in acquiring knowledge. It encompasses two primary approaches: supervised learning and unsupervised learning [16,17]. While unsupervised learning has gained recent attention and demonstrated promising results in various domains (e.g., object recognition, image segmentation, anomaly detection, image retrieval, image compression, image generation, etc.) [18,19,20,21], supervised learning remains pivotal in most deep learning work and applications. Crucial factors contributing to the extensive utilization of supervised learning include the availability of large-scale labeled datasets, task-specific learning, higher performance, broader applications, and higher interpretability. Advances in technology have facilitated the collection and annotation of massive amounts of data from various sources. These labeled datasets enable deep learning models to discern complex patterns and exhibit strong generalization capabilities when faced with new and unseen examples.

One of the most significant impacts of deep learning techniques has been in the field of computer vision. Incorporating deep learning has greatly influenced 3D reconstruction methods, leading to substantial advancements. Leveraging its ability to comprehend intricate patterns and representations from extensive datasets, deep learning has brought a transformative shift in 3D reconstruction. Its application spans different phases of the reconstruction workflow, encompassing fundamental feature learning and more complex tasks such as dense 3D reconstruction, shape completion, surface reconstruction, and single-view and multi-view reconstruction. Deep learning techniques can potentially optimize the efficiency of the process, enabling real-time or high-speed 3D reconstruction at a super-resolution level [22,23,24]. Various output representations can be employed in deep learning techniques for 3D object reconstruction, including volumetric representations, surface-based representations, and intermediate representations [25]. Sun et al. introduced a NeuralRecon framework for real-time scene reconstruction using a learning-based TSDF fusion module [26]. Additionally, Zhao et al. proposed a method that can accelerate the 3D reconstruction up to 10 Hz using fully-connected conditional random fields model [27]. To address computational cost and memory efficiency issues, a method named occupancy networks proposed a new representation for 3D output training with reduced memory footprint [28]. Three-dimensional reconstruction via deep learning has also found key applications in augmented reality (AR) tasks. For instance, Park et al. developed a smart and user-centric task assistance method that combines instance segmentation and deep learning-based object detection to reconstruct 2.5D and 3D replicas in wearable AR smart glasses [29]. In addition, 3D reconstruction through deep learning has been applied in various indoor mapping applications using mobile devices [30,31,32].

Deep learning has also emerged as an AI-assisted tool in the field of experimental mechanics and metrology, where precision is vital. It simplifies traditional techniques while ensuring consistent accuracy and allows real-time or high-speed measurements. In recent years, there has been a growing interest in integrating deep learning with the aforementioned structured-light technique, which is popular in a few fields, including optics, experimental mechanics, metrology, and computer vision, to achieve accurate 3D shape measurement and 3D reconstruction. This combination can substantially simplify and enhance conventional techniques while maintaining stable accuracy [33,34,35,36,37]. It holds promise for numerous scientific and engineering applications where accurate and efficient 3D reconstruction is paramount.

Among various structured light techniques, fringe projection profilometry (FPP) is the most widely used technique in combination with the deep learning method for 3D reconstruction [38,39,40,41,42]. The integrated approaches can be broadly categorized into fringe-to-depth and fringe-to-phase techniques. In the fringe-to-depth approach, a direct conversion of the captured fringe pattern(s) to the desired depth information is accomplished using convolutional neural networks (CNNs). This process is analogous to the image-to-image transformation in computer vision applications. By training CNN models on appropriate datasets, the fringe patterns can be effectively mapped to corresponding depth values, enabling accurate 3D reconstruction [43,44,45,46,47,48]. On the other hand, the fringe-to-phase approach exploits the multi-stage nature of the FPP. It involves transforming the fringe pattern(s) into intermediate results, which ultimately enable the acquisition of precise phase distributions. These phase distributions and camera calibration information are then utilized to achieve accurate 3D reconstruction [49,50,51,52,53,54,55].

In general, the fringe-to-phase approaches tend to yield more detailed 3D reconstruction results than the fringe-to-depth counterpart. This is primarily attributed to its incorporating additional phase calculations and utilizing parameter information obtained through camera calibration. Over the past few years, fringe-to-phase approaches, which focus on obtaining precise unwrapped phase distributions, have undergone notable developments in several aspects. These advancements include the employment of single or multiple input(s)/output(s), the introduction of reference planes, the implementation of multi-stage networks, the utilization of combined red-green-blue (RGB) color fringe images, and the use of coded patterns, among others [56,57,58]. Regardless of the specific variations, it is evident that the integration primarily relies on choosing single or multiple inputs. The subsequent training of the network(s) and the output(s) definition can be determined based on the researcher’s preferences and interests. In addition to several advanced fringe-to-phase techniques that utilize single-shot input and single network, alternative deep learning-based approaches have employed multi-shot inputs with multi-stage networks [59,60,61]. As an example, Yu et al. [62] introduced a concept where a single or two fringe patterns are transformed into multiple phase-shifted fringe patterns using multiple FTPNet networks. Liang et al. [63] utilized a similar autoencoder-based network in a two-step training process to derive the unwrapped phase from the segmented wrapped phase. In other studies, the researchers [57,64] employed two subnetworks with cosine fringe pattern and multi-code/reference pattern to obtain the wrapped phase and fringe orders. The work reported in [65,66] followed a framework comprising two deep neural networks, aiming to enhance the quality of the fringe pattern and accurately determine the numerator and denominator through denoising patterns. Machineni et al. [67] presented an end-to-end deep learning-based framework for 3D object profiling, and the method encompassed a two-stage process involving a synthesis network and a phase estimation network. Its notable drawbacks and limitations include the need for multiple training hardwares, extended training duration, a higher number of learning parameters, and a sequential process.

Drawing upon the advancements in single-shot 3D reconstruction techniques and recognizing the limitations of multi-stage multi-shot approaches, this paper presents a proof-of-concept 3D reconstruction method. The proposed approach utilizes a single network and employs a time-distributed wrapper to handle multiple inputs. The technique employs a time-distributed framework to convert multiple fringe images into intermediate results of numerators and denominators of arctangent functions, enabling the subsequent acquisition of phase distributions and 3D shape information. Unlike stacking multiple inputs and outputs in the spatial domain of the training vector, the proposed approach encodes multiple inputs and their corresponding outputs into temporal slices of the training vector. Similar to training and prediction using the spatial vector, the proposed framework can predict the intermediate results for unseen objects once the training process is successfully completed.

It should be emphasized that the classic FPP technique serves a dual purpose in this study. First, it prepares training data with ground-truth labels for the learning process. Second, it plays a crucial role in the subsequent process of obtaining the phase distributions and final 3D point cloud after the deep learning prediction. Given that the temporal FPP technique involves capturing multiple fringe images over a span of time, the proposed time-distributed framework is a well-suited approach for effectively handling and converting multiple inputs within the reconstruction process. The proposed technique brings several noteworthy contributions in comparison with previous fringe-to-phase methods:

1. It introduces a single network instead of relying on multiple subnetworks for a multi-stage process.
2. It presents a proof-of-concept 3D reconstruction approach where multiple inputs are stacked in the temporal domain vector rather than the spatial domain vector.
3. The data labeling process is simplified, with multiple inputs and corresponding outputs consolidated into a single training vector instead of separate vectors.
4. It maintains the accuracy advantages of the classic FPP method while reducing the number of required fringe patterns.

The remaining sections of this paper are structured as follows. Section 2 provides an overview of the FPP technique and presents the proposed framework for phase measurement. In Section 3, various experiments are conducted to assess the effectiveness of the proposed approach. Section 4 presents discussions and further analysis of the results, while Section 5 offers a concise summary of the proposed work.

## 2. Materials and Methods

The process of FPP 3D imaging involves two main steps. First, evenly spaced fringe patterns are projected onto the surface of the target, and the surface profile is encoded in the distorted fringe patterns. A camera then captures the patterns for subsequent 3D decoding. This decoding process comprises four key sub-steps: phase extraction, phase unwrapping, depth determination, and 3D reconstruction. It is worth noting that the proposed time-distributed framework specifically focuses on converting fringe-pattern images into their corresponding numerators and denominators in the phase determination function. Nevertheless, it should also be emphasized that the subsequent phase determination and 3D reconstruction still rely on the conventional FPP technique. Therefore, providing an overview of the classic FPP technique is essential before discussing the proposed time-distributed network.

### 2.1. Temporal Structured-Light Technique: Fringe Projection Profilometry

The temporal-based FPP technique involves projecting a series of fringe patterns onto the surface of the target object. The fringe patterns used in this technique can be described as uniform, with consistent characteristics across the entire projection:

(1) $$I_{j}^{i}\left(u,v\right)=I_{0}+I_{0}\cos\left(\phi_{i}\left(u,v\right)+\delta_{j}\right)$$

where *I* represents the intensity of the projected input at a specific pixel location (u,v); the subscript *j* denotes the order of the phase-shifted image, with *j* ranging from 1 to 4 in the case of a four-step phase-shifting algorithm; and superscript *i* implies the *i*th frequency. The intensity modulation is represented by the constant value $I_{0}$, typically set to 127.5. The fringe phase ϕ can be expressed as $\phi_{i}\left(u,v\right)=2\pi f_{i}\frac{u}{W}$, where $f_{i}$ corresponds to the fringe frequency defined as the number of fringes in the entire pattern, and *W* represents the width of the pattern. Moreover, the phase-shift amount δ is given by $\delta_{j}=\frac{(j-1)\pi }{2}$.

In practice, the fringe patterns captured from the synchronous camera are distinct from the generated fringe patterns and can be elaborated as follows [68]:

(2) $$I_{j}^{i}\left(u,v\right)=I_{a}\left(u,v\right)+I_{b}\left(u,v\right)\cos\left[\phi_{i}\left(u,v\right)+\delta_{j}\right]$$

where *I*, $I_{a}$, and $I_{b}$ represent the pixel intensities of the captured patterns, the intensity background, and the fringe amplitude at a specific pixel location (u,v). The value of $\phi_{i}\left(u,v\right)$ can be computed using the standard phase-shifting algorithm. In this study, we utilize the four-step phase-shifting algorithm, and the determination of $\phi_{i}^{w}\left(u,v\right)$ is given by the following equation [69]:

(3) $$\phi_{i}^{w}\left(u,v\right)=\arctan\frac{I_{4}^{i}\left(u,v\right)-I_{2}^{i}\left(u,v\right)}{I_{1}^{i}\left(u,v\right)-I_{3}^{i}\left(u,v\right)}=\arctan\frac{N^{i}}{D^{i}}$$

where *N* and *D* denote the numerator and denominator of the arctangent function, respectively. Hereinafter, the pixel coordinate (u,v) will be omitted to streamline the subsequent equations. The result obtained from Equation (3) lies within the range of [−π,π), and to obtain the true phase, it is necessary to unwrap $\phi_{i}^{w}$. In the context of FPP 3D imaging, the multi-frequency phase-shifting algorithm is widely recognized for its ability to handle geometric discontinuities and situations involving overlapping objects with varying height or depth information.

In our proposed approach, we utilize the dual-frequency four-step (DFFS) phase-shifting scheme, which involves two frequencies ($f_{1}$ and $f_{2}$), as well as the triple-frequency four-step (TFFS) scheme, which incorporates three frequencies ($f_{1}$, $f_{2}$, and $f_{3}$). These schemes are employed to obtain high-quality unwrapped phase maps and serve as the ground-truth labels for training the proposed time-distributed network.

**Figure 2:**
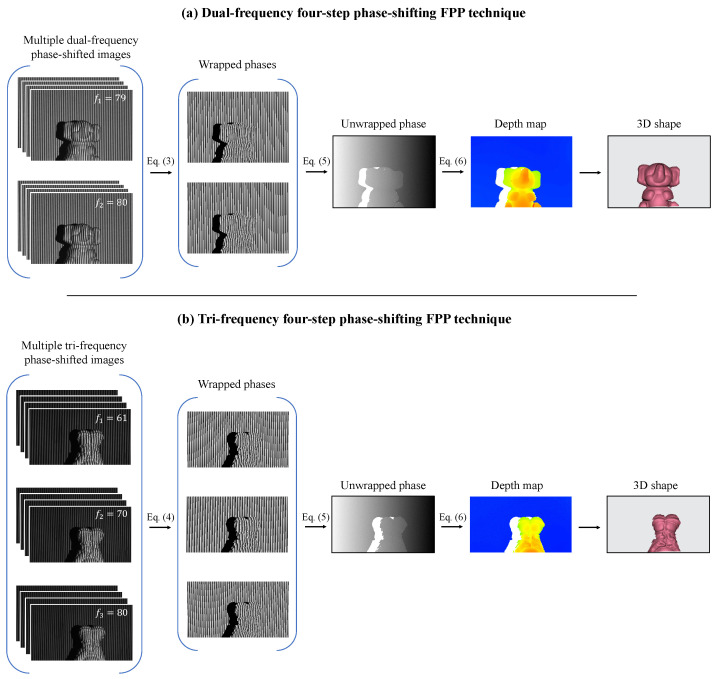
Flowchart of the FPP 3D imaging technique with DFFS (**a**) and TFFS (**b**) phase-shifting schemes.

When using the DFFS phase-shifting scheme, the unwrapped phase can be obtained by satisfying the condition $f_{2}-f_{1}=1$. In such cases, the equations governing the unwrapped phase can be expressed as follows [70]:

(4) $$\begin{array}{cc} \phi_{12}^{uw} & =\phi_{2}^{w}-\phi_{1}^{w}+\left\{\begin{array}{cc} 0, & \phi_{2}^{w}\geq \phi_{1}^{w}\\ 2\pi , & \phi_{2}^{w}<\phi_{1}^{w} \end{array}\right.\\ \phi & =\phi_{2}^{uw}=\phi_{2}^{w}+\mathrm{INT}\left(\frac{\phi_{12}^{uw}f_{2}-\phi_{2}^{w}}{2\pi }\right)2\pi \end{array}$$

where $\phi_{1}^{w}$ and $\phi_{2}^{w}$ are the wrapped phases of two frequencies $f_{1}$ and $f_{2}$, respectively. The initial unwrapped phase, $\phi_{12}^{uw}$, is derived from the pattern with only one fringe. However, due to the noise caused by the frequency mismatch between $f_{1}$ and $f_{2}$, $\phi_{12}^{uw}$ cannot be directly used. Instead, it serves as the interfering unwrapped phase for the hierarchical phase-unwrapping process of $\phi_{2}^{uw}$. The final unwrapped phase, denoted as ϕ, corresponds to the phase distribution of the highest fringe frequency. This study utilizes two frequencies, $f_{1}=79$ and $f_{2}=80$, in accordance with the requirements of the DFFS scheme. Figure 2a illustrates the flowchart of the DFFS phase-shifting scheme.

In the TFFS scheme, as depicted in Figure 2b, if the three frequencies fulfill the condition $\left(f_{3}-f_{2}\right)-\left(f_{2}-f_{1}\right)=1$, where $\left(f_{3}-f_{2}\right)>\left(f_{2}-f_{1}\right)>0$, the unwrapped phase of the fringe patterns with the highest frequency can be computed using the following hierarchical equations [71,72]:

(5) $$\begin{array}{cc} \phi_{12}^{w} & =\phi_{2}^{w}-\phi_{1}^{w}+\left\{\begin{array}{cc} 0 & \;\phi_{2}^{w}⩾\phi_{1}^{w}\\ 2\pi & \;\phi_{2}^{w}<\phi_{1}^{w} \end{array}\right.\\ \phi_{23}^{w} & =\phi_{3}^{w}-\phi_{2}^{w}+\left\{\begin{array}{cc} 0 & \;\phi_{3}^{w}⩾\phi_{2}^{w}\\ 2\pi & \;\phi_{3}^{w}<\phi_{2}^{w} \end{array}\right.\\ \phi_{123} & =\phi_{23}^{w}-\phi_{12}^{w}+\left\{\begin{array}{cc} 0 & \;\phi_{23}^{w}⩾\phi_{12}^{w}\\ 2\pi & \;\phi_{23}^{w}<\phi_{12}^{w} \end{array}\right.\\ \phi_{23} & =\phi_{23}^{w}+\mathrm{INT}\left(\frac{\phi_{123}\left(f_{3}-f_{2}\right)-\phi_{23}^{w}}{2\pi }\right)2\pi \\ \phi & =\phi_{3}^{uw}=\phi_{3}^{w}+\mathrm{INT}\left(\frac{\phi_{23}\frac{f_{3}}{f_{3}-f_{2}}-\phi_{3}^{w}}{2\pi }\right)2\pi \end{array}$$

where ϕ with superscript *w* and uw are the wrapped phase and unwrapped phase, respectively. The function “INT” rounds the value to the nearest integer. The term $\phi_{mn}$ represents the difference between $\phi_{m}$ and $\phi_{n}$, where $\left(f_{n}-f_{m}\right)$ corresponds to the number of wrapped fringes in the phase map. The algorithm’s core principle is based on the fact that $\phi_{123}$ is both wrapped and unwrapped due to the presence of only one fringe in the pattern. This property enables a hierarchical phase-unwrapping process that connects $\phi_{123}$ and $\phi_{3}$ through $\phi_{23}$. The phase distribution of the highest-frequency fringe patterns, $\phi_{3}$, is utilized for the final phase determination as it provides the highest level of accuracy. In the TFFS scheme, the chosen frequencies are 61, 70, and 80. These specific frequencies were selected to maintain a balanced hierarchical calculation with a ratio of 1:10:80.

Ultimately, the FPP 3D imaging technique is employed to directly reconstruct the height/depth information from the unwrapped phase obtained from Equation (4) or Equation (5). The equation governing the retrieval of the depth map from ϕ can be derived as described in [69]:

(6) $$\begin{array}{cc} z & =\frac{c{\left[\begin{array}{c} P_{1}\;P_{2} \end{array}\right]}^{⊺}}{d{\left[\begin{array}{c} P_{1}\;P_{2} \end{array}\right]}^{⊺}}\\ c & =\left\{1\;\;c_{1}\;\;c_{2}\;\;c_{3}\;\;\cdots \;\;c_{17}\;\;c_{18}\;\;c_{19}\right\}\\ d & =\left\{d_{0}\;\;d_{1}\;\;d_{2}\;\;d_{3}\;\;\cdots \;\;c_{17}\;\;d_{18}\;\;d_{19}\right\}\\ P_{1} & =\left\{1\;\;\phi \;\;u\;\;u\phi \;\;v\;\;v\phi \;\;u^{2}\;\;u^{2}\phi \;\;uv\;\;uv\phi \;\;v^{2}\;\;v^{2}\phi \right\}\\ P_{2} & =\left\{u^{3}\;\;u^{3}\phi \;\;u^{2}v\;\;u^{2}v\phi \;\;uv^{2}\;\;uv^{2}\phi \;\;v^{3}\;\;v^{3}\phi \right\}. \end{array}$$

The equation for determining the height or depth value *z* at a specific pixel coordinate (u,v) involves using triangulation parameters. These parameters, denoted as $c_{1}$ to $c_{19}$ and $d_{0}$ to $d_{19}$, are obtained through a system calibration process.

This study used a set of 31 sculptures showing various surface shapes, as well as 10 objects commonly found in laboratories, including gauge block, tape measure, corded telephone, remote control, ping-pong ball, electronic charger, glue bottle, calibration board, rotary fan, and balloon [33]. Each object was arbitrarily positioned many times in the field of view to serve as multiple different targets. In addition, two or multiple objects were randomly grouped together to form new objects for the dataset generation.

**Figure 3:**
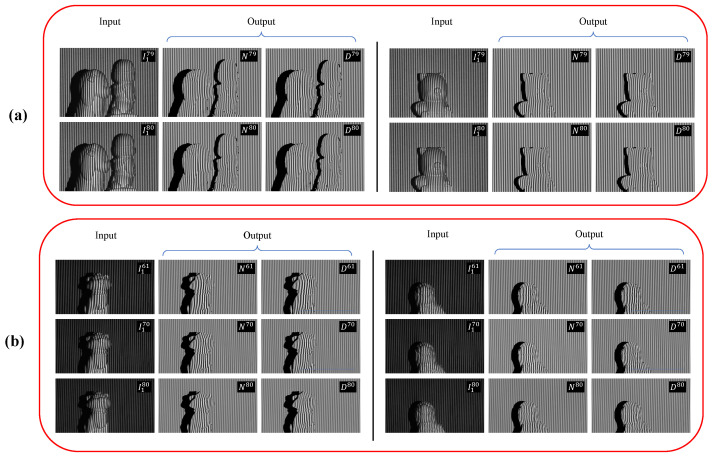
Examplars of input–output pair in (**a**) DFFS datasets and (**b**) TFFS datasets.

The DFFS datasets consisted of a total of 2048 scenes with a resolution of 640×448 [39,70]. Each scene involved the projection of 8 uniform sinusoidal four-step phase-shifted images, with two frequencies of $f_{1}=79$ and $f_{2}=80$, by the projector. Simultaneously, the camera captured 8 corresponding images. During the data labeling process, the first image of each frequency, namely $I_{1}^{79}$ and $I_{1}^{80}$, was selected as the temporal input slices. The corresponding output of numerators and denominators, represented as $N^{79}$, $D^{79}$, $N^{80}$, and $D^{80}$, was generated using all 8 captured images and Equation (3). Figure 3a illustrates examples of the input–output pairs used for the proposed time-distributed framework with the DFFS datasets.

Likewise, the TFFS datasets consisted of 1500 data samples with the resolution of 640×352 [71,72], with each scene capturing a total of 12 images. These four-step phase-shifted images employed three frequencies: $f_{1}=61$, $f_{2}=70$, and $f_{3}=80$. Figure 3b shows two examples of input–output pairs generated for the TFFS datasets.

### 2.2. Time-Distributed Framework for Temporal Fringe-Pattern Transformation

The primary aim of the proposed time-distributed (TD) framework remains consistent with previous fringe-to-phase approaches, focusing on the determination of phase distributions for 3D shape measurement. However, the specific goal of this framework is to showcase a proof-of-concept image-to-image conversion using deep learning techniques for the temporal FPP technique.

Time-distributed is a term commonly employed in Recurrent Neural Networks (RNNs) or sequence-to-sequence models, where it is utilized in the context of sequential data, such as a sequence of images. In the context of the temporal FPP technique, which involves multiple fringe patterns captured at different time steps, the time-distributed concept allows using the same network parameters (weights and biases) to process each individual input separately. This ensures that the network can extract consistent features, such as phase-shifted information, from each time step while facilitating the learning of temporal dependencies, such as consecutive frequencies.

**Figure 4:**
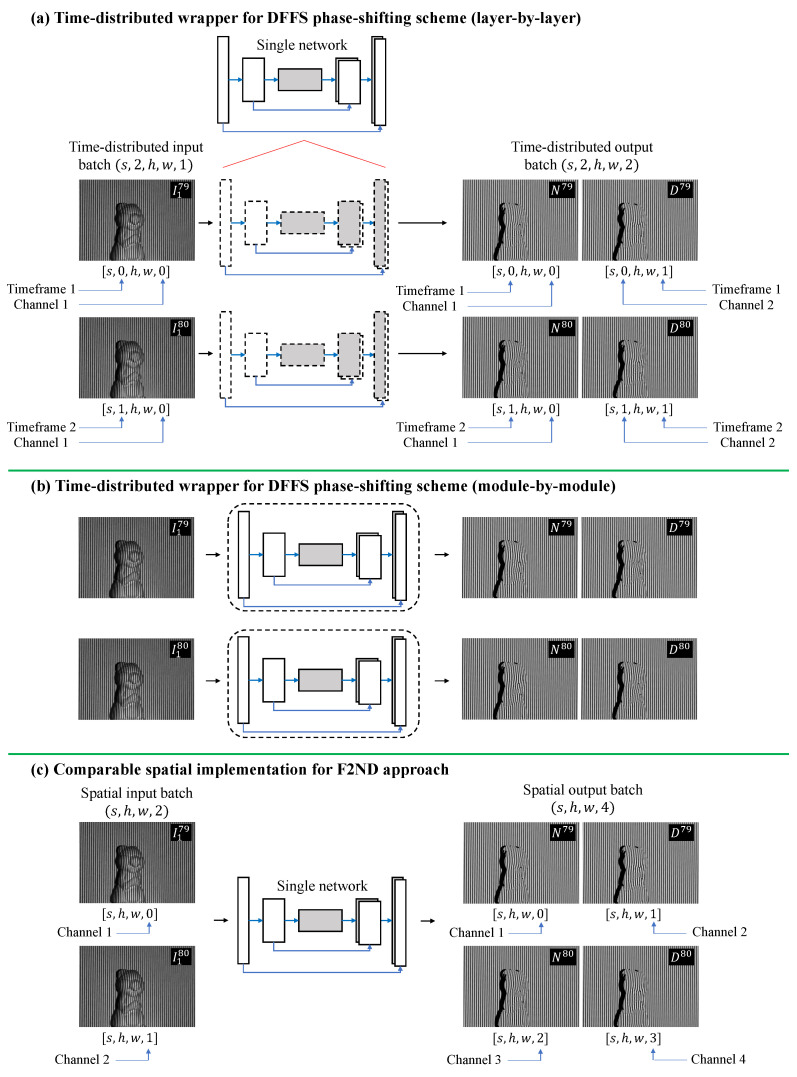
(**a**,**b**) Time-distributed concept for DFFS phase-shifting scheme, and (**c**) the comparable spatial F2ND approach.

**Figure 5:**
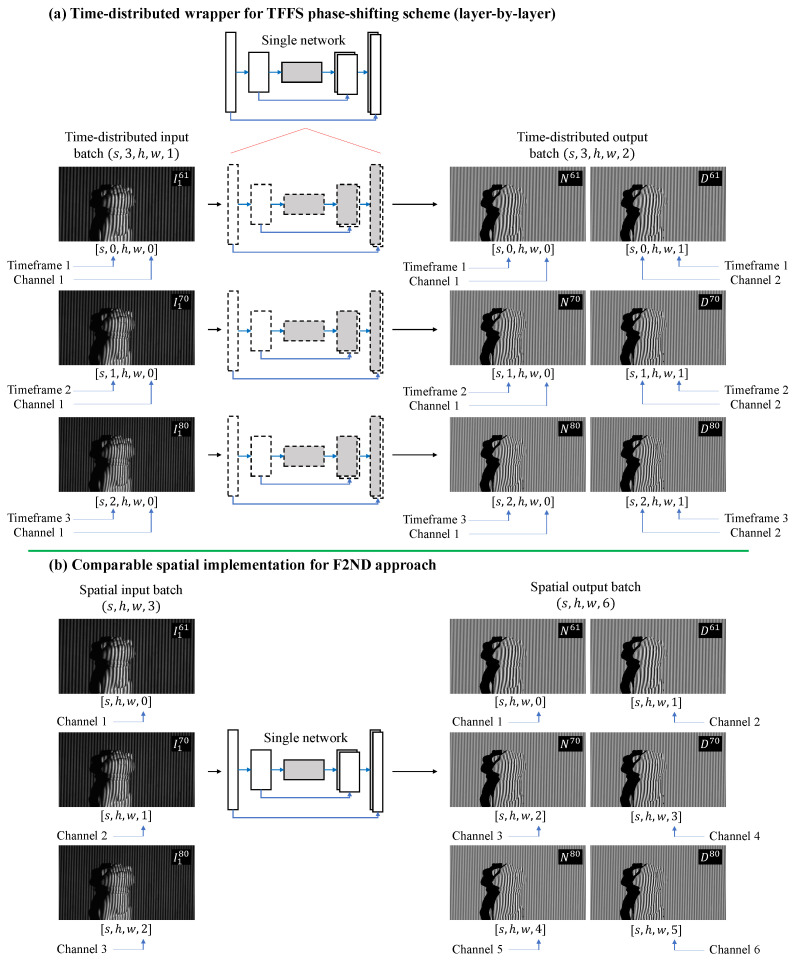
(**a**) Time-distributed concept for TFFS phase-shifting scheme, and (**b**) the comparable spatial F2ND approach.

Figure 4a and Figure 5a present the workflow of the proposed TD framework, which is specifically designed for converting sequential fringe-to-phase data. The goal of this framework is to train the model to convert the given fringe patterns into their corresponding phase-shifted information, namely the numerators and denominators. However, unlike the conventional approach that combines all spatial and temporal information in the spatial domain, as depicted in Figure 4c and Figure 5b, the TD framework differentiates and distributes the spatial and temporal information into two distinct learning concepts. The first concept involves extracting features, such as performing the fringe-to-ND (F2ND) conversion, for each individual frame within the time steps. This is illustrated by each row or the horizontal direction in the figures. The second concept focuses on applying the same feature extraction process to consecutive temporal frequencies represented in the vertical direction. By segregating and distributing the spatial and temporal information in this manner, the TD framework enables effective and efficient learning of the desired features.

In this study, the TD framework utilizes a widely used network architecture called UNet for image-to-image conversion [73]. The network consists of an encoder and a decoder path with symmetric concatenation for accurate feature transformation. The encoder path employs ten convolution layers and four max-pooling layers, reducing the resolution but increasing the filter depth. The decoder path includes eight convolution layers and four transposed convolution layers, enriching the input feature maps to higher resolution while decreasing the filter depths. A 1×1 convolution layer at the end of the decoder path leads to the numerator and denominator outputs. The proposed framework employs a linear activation function and mean-squared error (MSE) loss for training, considering the continuous nature of the output variables. Details of the network architecture are explained in detail in our previous works [70,71,72].

In Figure 4 and Figure 5, **the TD framework utilizes a single network**, where the same weights and biases are applied for feature extraction across the temporal slices. The dashed line in these figures represents the TD concept. Two approaches for implementing the TD concept in the deep learning network are introduced: TD Layer and TD Module. In the TD Layer approach, the TD wrapper is applied to each layer of the learning model, as shown in Figure 4a and Figure 5a. The TD wrapper encapsulates the entire network model in the TD Module approach, as depicted in Figure 4b. Although the F2ND conversion task remains the same, it is valuable to investigate the framework’s performance using different implementations. In Keras implementation, the TD Layer and TD Module can be better understood through the following examples:

- **TD Layer**
  output = keras.layers.TimeDistributed(keras.layers.Conv2D(…))(input)
- **TD Module**
  module = keras.Model(network_input, network_output)
  output = keras.layers.TimeDistributed(module)(input)

To compare the performance of the framework with previous methods using the spatial domain, a popular spatial F2ND approach is employed, where all the input and output data are organized in the spatial slices, as shown in Figure 4c and Figure 5b. The input–output pair selected for this framework is a commonly used combination in the field. The input consists of consecutive fringe patterns captured at different time steps, each with a distinct frequency. The corresponding output comprises the numerators and denominators associated with these fringe patterns.

The preparation of multidimensional data format for the TD network differs from that of a regular spatial convolution network. In the TD network, the input, output, and internal hidden layers are represented as five-dimensional tensors with shapes $\left(s,t,h,w,c\right)$, where *s* indicates the number of data samples, *t* denotes the timeframe of each different frequency, *h* and *w* represent the height and width of the input, output, or feature maps at the sub-scale resolution layer, respectively, and *c* is the channel or filter depth. In this study, *t* is set as 2 and 3 for the DFFS and TFFS schemes, respectively. Moreover, *c* is set to 1 for the input of a single grayscale image and 2 for the output of the numerator and denominator at each timestep. Clear visualization of this multidimensional data is explained in detail and depicted in Figure 4 and Figure 5.

**Hyperparameter tunning**: The convolution layers are employed with a LeakyReLU function, introducing a small negative coefficient of 0.1 to address the zero-gradient problem. Additionally, a dropout function with a rate of 0.2 is incorporated between the encoder and the two decoder paths to enhance robustness. The model is trained for 1000 epochs with a mini-batch size of 2, using the Adam optimizer with an initial learning rate of 0.0001 for the first 800 epochs. Afterward, a step decay schedule is implemented to gradually reduce the learning rate for better convergence [74]. To prevent overfitting, various data augmentation techniques, including ZCA whitening, brightness, and contrast augmentation, are employed. During training, the mean squared error (MSE) is used as the evaluation metric, and Keras callbacks like History and ModelCheckpoint are utilized to monitor training progress and save the best model.

## 3. Experiments and Results

The performance of the proposed TD framework was evaluated through a range of quantitative and qualitative analyses. Firstly, the quantitative assessment included using two image quality metrics, namely Structural Similarity Index Measure (SSIM) and Peak Signal-to-Noise Ratio (PSNR), to evaluate the predicted numerators and denominators. Additionally, four error metrics and three accuracy metrics were employed to verify the depth accuracy of the proposed technique. Secondly, qualitative comparisons were made by visually examining the 3D shape reconstructions of test objects generated using the TD Layer, TD Module, and a comparable F2ND approach. These analyses provided a comprehensive evaluation of the performance of the proposed TD framework.

The datasets were captured using an RVBUST RVC-X mini 3D camera (Figure 1b), which provides an ideal camera–projector–target triangulation setup. The training process utilized multiple GPU nodes available in the Biowulf cluster of the High-Performance Computing group at the National Institutes of Health. The main GPUs used were 4 × NVIDIA A100 GPUs with 80 GB VRAM and 4 × NVIDIA V100-SXM2 GPUs with 32 GB VRAM. To optimize performance, Nvidia CUDA Toolkit 11.2.2 and cuDNN v8.1.0.77 were installed on these units. The network architecture was constructed using TensorFlow v2.8.2 and Keras v2.8.0, popular open-source deep learning frameworks and Python libraries known for their user-friendly nature.

### 3.1. Quantitative Evaluation of TD Layer, TD Module, and Spatial F2ND in DFFS and TFFS Datasets

Upon the completion of training in the TD framework, the predicted numerators and denominators are further processed using the classic FPP technique to derive the unwrapped phase distributions and 3D depth/shape information. It is important to note that the TD framework’s primary task is converting fringe patterns to their corresponding numerators or denominators, also known as the F2ND conversion or image-to-image conversion. To quantitatively evaluate the accuracy of the reconstructed numerators and denominators, SSIM and PSNR metrics were utilized. These metrics provide valuable insights into the similarity and fidelity of the reconstructed results, enabling a quantitative evaluation of the performance of the TD framework.

**Figure 6:**
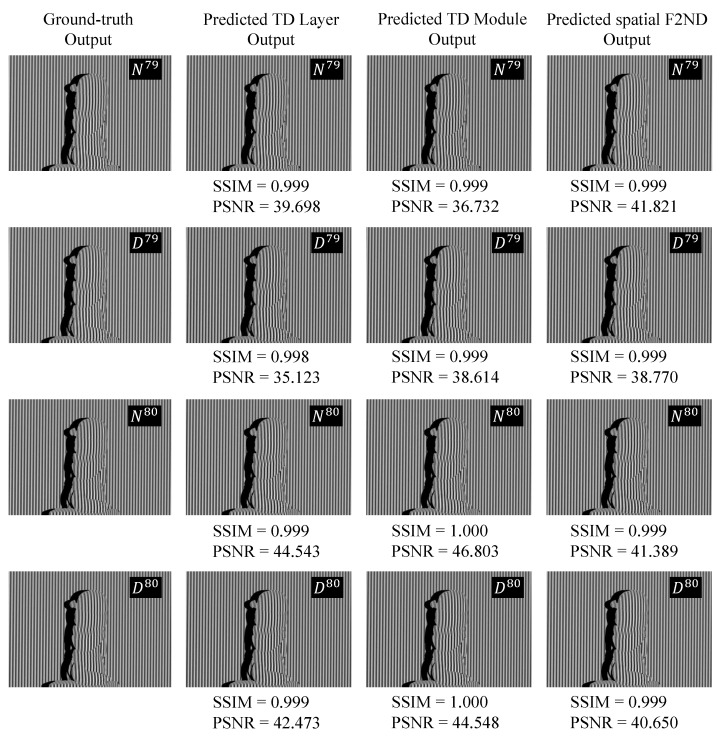
Evaluation of image quality metrics (SSIM and PSNR) for predicted numerators and denominators.

Figure 6 showcases the predicted output of an unseen test object utilizing the DFFS datasets, accompanied by the corresponding evaluation metrics. Upon careful examination, it may initially appear challenging to visually discern any noticeable disparities between the predicted numerators/denominators and the ground truth counterparts. However, an in-depth analysis of the structural similarity index (SSIM), ranging from 0.998 to 1.000, and the peak signal-to-noise ratio (PSNR), which consistently hovers around 40, provides valuable insights. These metrics collectively suggest that the reconstructed images resemble the reference ground-truth images, affirming their high degree of fidelity and accuracy. The TD framework demonstrates comparable performance to the spatial F2ND approach, confirming its effectiveness in capturing spatial information for accurate predictions.

The depth measurement accuracy is an essential quantitative measure for evaluating the FPP 3D imaging technique. In this study, various error and accuracy metrics commonly employed for assessing monocular depth reconstruction are utilized. These metrics are calculated by comparing the predicted depth map with the ground-truth depth map. The proposed TD Layer, TD Module, and the spatial F2ND approach are subjected to quantitative evaluation using these metrics in both DFFS and TFFS datasets. The evaluation encompasses four error metrics and three accuracy metrics, which provide a comprehensive assessment of the performance of the different approaches:

- Absolute relative error (rel): $\frac{1}{n}\sum_{i=1}^{n}\frac{\left|\hat{z_{i}}-z_{i}\right|}{\hat{z_{i}}}$
- Root-mean-square error (rms): $\sqrt{\frac{1}{n}\sum_{i=1}^{n}{\left(\hat{z_{i}}-z_{i}\right)}^{2}}$
- Average $log_{10}$ error (log): $\frac{1}{n}\sum_{i=1}^{n}\left|\begin{array}{c} log_{10}\left(\hat{z_{i}}\right)-log_{10}\left(z_{i}\right) \end{array}\right|$
- Root-mean-square log error (rms log):
  $\sqrt{\frac{1}{n}\sum_{i=1}^{n}\left|\begin{array}{c} log_{10}\left(\hat{z_{i}}\right)-log_{10}\left(z_{i}\right) \end{array}\right|}$
- Threshold accuracy: $\delta =(\frac{\hat{z_{i}}}{z_{i}},\frac{z_{i}}{\hat{z_{i}}})<thr;$
  $thr\in \left\{\begin{array}{c} 1.25,1.25^{2},1.25^{3} \end{array}\right\}$

where $\hat{z_{i}}$ and $z_{i}$ represent the ground-truth depth determined in Equation (6) and the predicted depth at valid *i*th pixel, respectively. The key quantitative analyses are presented in Table 1. Upon examining the DFFS datasets, it is evident that the spatial F2ND approach demonstrates slightly superior performance compared with the proposed TD Layer and TD Module approaches. Nevertheless, the differences in performance are negligible as all the metrics exhibit similar values. Notably, the TD Layer and TD Module approaches outperform the spatial F2ND approach in the TFFS datasets, as observed in the error and accuracy metrics. These quantitative metrics provide evidence that the proposed techniques not only serve as a proof of concept but also yield comparable or slightly improved results compared with the state-of-the-art techniques used in previous studies.

To ascertain the distinctions among the proposed TD Layer, TD Module, and spatial F2ND approaches in terms of accuracy, additional statistical analyses were performed. The non-parametric Kruskal–Wallis H-test was selected for this task, utilizing the mean absolute error (MAE) values as test samples. These MAE values represent the disparities between the ground-truth depths and the predicted depths generated by each approach (TD Layer, TD Module, and spatial F2ND).

The outcomes of the Kruskal–Wallis H-test revealed significant error differences among the three groups for both the DFFS dataset (H = 8.532, p = 0.014) and the TFFS dataset (H = 21.144, p = 1.029 ×$10^{-9}$). This statistical analysis provides evidence of the notable variations in accuracy between the three approaches.

### 3.2. 3D Reconstruction from DFFS Phase-Shifting Scheme via Time-Distributed Concept

Visual comparisons of the 3D shape surfaces were conducted to further assess the proposed techniques’ performance. The depth maps obtained from the ground truth, TD Layer, TD Module, and the comparable spatial F2ND approach were analyzed for differences. This visual evaluation provides additional insights into the accuracy and quality of the reconstructed 3D shape surfaces.

**Figure 7:**
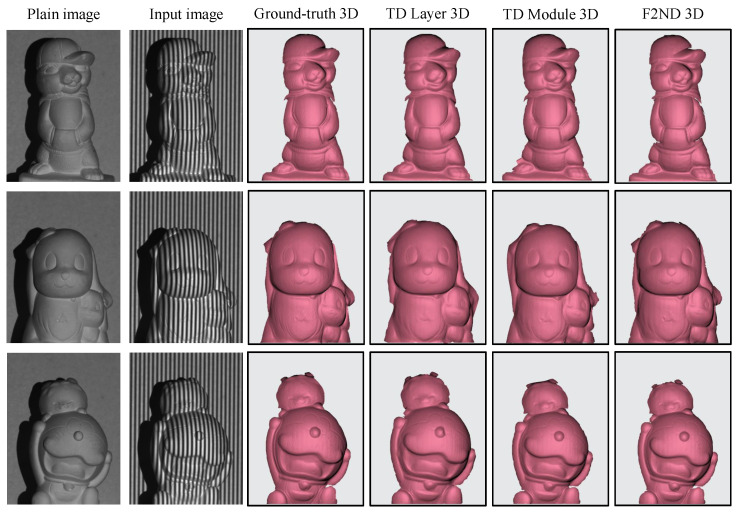
3D shape reconstruction of a single-object scene using DFFS datasets.

The 3D reconstruction of three different objects is showcased in Figure 7, with each object corresponding to a single scene. The first and second columns of the figure display the original image and an example input image, respectively. The subsequent four columns present the 3D reconstructions obtained from the ground truth, TD Layer, TD Module, and the comparable spatial F2ND approach. It should be noted that the scenes have been cropped and zoomed in to enhance visibility and facilitate a comparative analysis of the results. Upon visual inspection of the figure, it is evident that all three comparable techniques exhibit a high degree of similarity to the ground truth, with no significant degradation in the quality of the reconstructed results. However, a closer examination reveals that the 3D reconstruction outcomes obtained using the TD Layer exhibit a certain level of blurring, resulting in less detailed representations. Conversely, the spatial F2ND approach demonstrates more intricate joint structures in the reconstructed 3D surfaces. This observation aligns with the quantitative findings presented in Table 1, where the spatial F2ND approach demonstrates slightly superior performance.

**Figure 8:**
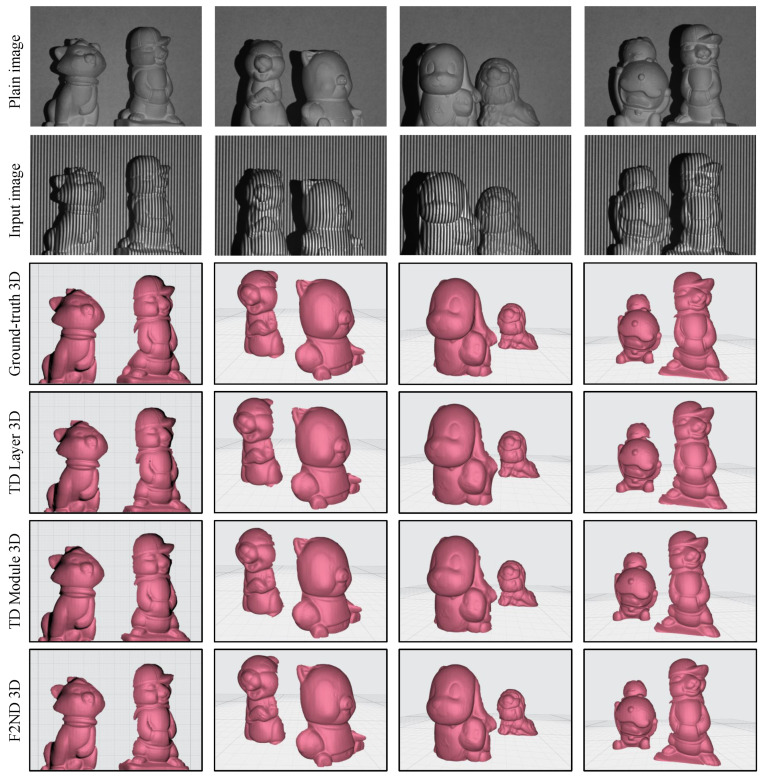
3D shape reconstruction of a scene with multiple objects using DFFS datasets.

Furthermore, the visual evaluation involves the reconstruction of scenes with multiple objects. The first two rows of Figure 8 showcase four scenes, each featuring distinct objects with varying heights and depths. It is worth mentioning that in the traditional FPP technique, obtaining continuous phase map distributions for separated objects poses a challenge due to the presence of discontinuous fringe order. The shadows in the background of the scenes provide valuable visual cues for observing the differences in depth between the objects, which contribute to the challenges associated with determining phase distributions and fringe order ambiguity. The reconstruction of the scenes reaffirms that both the TD Module and spatial F2ND approaches offer more detailed results than the TD Layer approach while maintaining overall similarity in terms of the shapes. To enhance the visibility of depth differences among the subjects, the grid pattern and view angle were adjusted during the scene reconstruction process.

### 3.3. 3D Reconstruction from TFFS Phase-Shifting Scheme via Time-Distributed Concept

**Figure 9:**
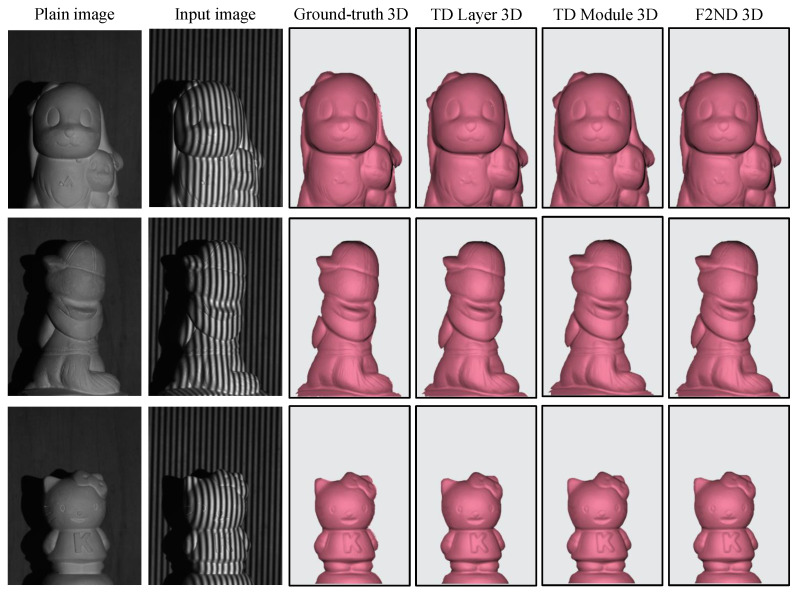
3D shape reconstruction of a single-object scene using TFFS datasets.

The TFFS datasets were utilized to evaluate the efficacy and feasibility of the proposed techniques in terms of 3D reconstruction. The 3D reconstruction of various techniques for a single object is depicted in Figure 9. At first glance, the reconstructed results closely resemble the ground truth, making it challenging to discern any notable differences. The reconstructed scenes exhibit similar shapes and depth information, suggesting that these techniques can accurately capture the underlying 3D structure. However, upon closer examination, the TD Layer technique stands out for its ability to capture finer details, particularly in the contoured and concave regions of the shape. This indicates that the TD Layer approach excels in preserving intricate features, resulting in a more faithful representation of the object’s surface.

**Figure 10:**
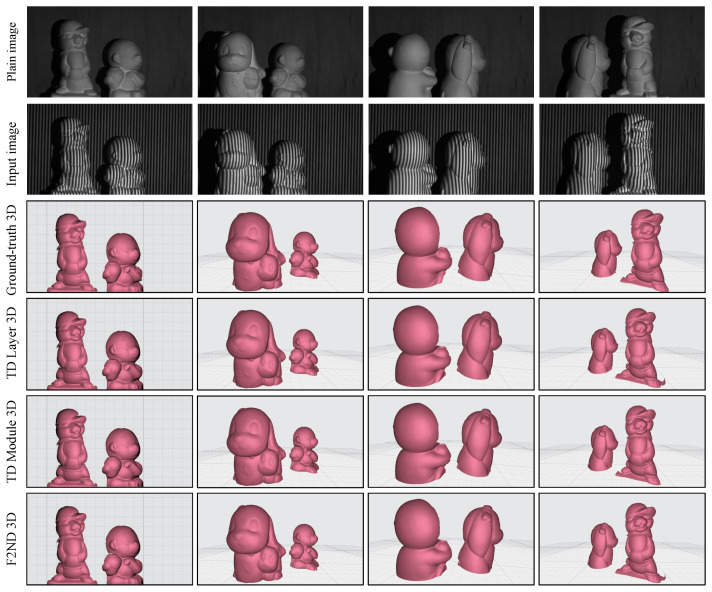
3D shape reconstruction of a scene with multiple objects using TFFS datasets.

Subsequently, the 3D reconstruction process was extended to encompass four distinct unseen scenes, each featuring multiple objects. The scenes were carefully configured from various angles to accentuate the differences in depth among the objects, a characteristic that is further emphasized by the presence of shadowed backgrounds. The obtained results in Figure 10 reveal that, while some minor discrepancies and variations near the object edges are observed, the reconstructed objects’ overall shape and intricate details are largely preserved and closely resemble the ground truth 3D representations. Despite the inherent challenges associated with accurately capturing depth information and intricate object surfaces, the proposed techniques effectively capture the main features and structures, demonstrating their ability to provide reliable and faithful 3D reconstructions.

## 4. Discussion

This paper explores the novel concept of a time-distributed wrapper to integrate the FPP technique with deep learning, specifically focusing on the F2ND transformation. The performance of the proposed approach is evaluated through comprehensive quantitative and qualitative analyses using TFFS and DFFS datasets. These analyses encompass comparisons of image quality, depth differences, and the visual appearance of the 3D reconstructions.

Overall, the proposed TD Layer and TD Module approaches demonstrate promising performance in terms of both quantitative measures and visual assessments. While the spatial F2ND technique may show slightly better results in certain quantitative metrics, the differences are marginal. The visual comparisons reveal that the proposed TD techniques can accurately capture the shapes and depth information of the objects, although the TD Layer technique may exhibit some blurring effects. These findings indicate that the TD Layer and TD Module approaches are viable alternatives to the traditional spatial F2ND technique, offering competitive performance in 3D reconstruction tasks.

It should be noted that alternative output vectors, such as multiple phase-shifted fringe images or wrapped phases with different frequencies, can be used instead of numerators and denominators. However, recent studies [39,70,71,75] have demonstrated that the spatial F2ND approach yields similar results to the fringe-to-fringe approach while requiring less storage space due to fewer channels in the output vector. Moreover, the fringe-to-wrapped phase approach is not considered ideal as it produces inferior results compared with the spatial F2ND approach.

Despite introducing the new concept of the time-distributed wrapper for the temporal FPP technique, the manuscript also acknowledges certain drawbacks and limitations. One limitation arises from the requirement of equal depth channels in both the input and output vectors. The time-distributed network cannot be trained if the depth channels differ across different timeframes. For instance, in the DFFS dataset, the first temporal output slice includes both numerators and denominators (i.e., [s,0,h,w,0] and [s,0,h,w,1]). In contrast, the second temporal output slice only consists of a single fringe order map [75] or a single coarse map [39] (i.e., [s,0,h,w,0]), resulting in a missing channel in the second temporal output slice.

**Figure 11:**
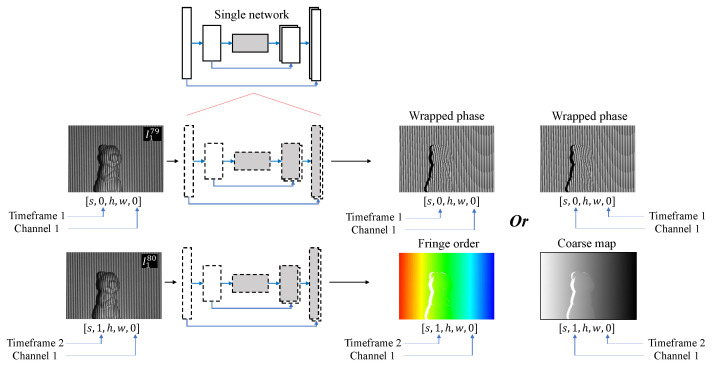
Potential application of TD framework with different output formats in FPP technique.

The previously mentioned limitation raises a question regarding the possibility of utilizing different output formats in the proposed approach of the TD framework. The answer is affirmative, provided that the depth channels in both the input and output vectors are consistent. Figure 11 showcases a potential application of the TD framework, where different output formats in the FPP technique are employed. The figure illustrates that the channel depth balance in the temporal slice remains at 1, utilizing either the pair of wrapped phase and fringe order or the pair of wrapped phase and coarse map. However, as stated earlier, using the wrapped phase typically leads to poor 3D reconstruction outcomes. Hence, it has been excluded from this investigation.

Although the proposed technique may not have been able to perform extensive comparisons with other well-established 3D reconstruction methods in diverse fields like image processing and computer vision, it has successfully carved out a unique niche in the narrower domain of optics and experimental mechanics. Notably, integrating the Fringe Projection technique and deep learning sets this approach apart as a novel and innovative 3D reconstruction technique, overcoming the limitations and weaknesses of previous multi-stage and multi-network approaches.

Moreover, the application of TimeDistributed Layer in this specific field is relatively scarce, highlighting the significance of our proposed technique as a pioneering example for a simple yet essential task such as image-to-image transformation. By showcasing the potential of the TimeDistributed concept, our work can inspire further exploration and adoption of this technique in various other fields, ultimately contributing to advancing 3D reconstruction and deep learning applications. One compelling application for the TimeDistributed Layer lies in reconstructing dynamic augmented reality (AR) views, incorporating time-oriented data. Leveraging the overlapping four-dimensional (4D) representations at different time viewpoints can effectively address occlusion issues in the real scene, resulting in improved and comprehensive visualizations [76,77]. Moreover, the TimeDistributed Layer shows promise in determining camera motion and pose for feature tracking in AR applications, enabling incremental motion estimates at various points in the time series [78,79]. Another intriguing use case is AR-based 3D scene reconstruction via the structure from motion (SFM) technique, which establishes relationships between different images [80,81]. These applications exemplify the versatility and potential of the TimeDistributed Layer, indicating its relevance beyond the specific field of 3D shape reconstruction.

Future research could focus on refining the TD techniques to address the minor discrepancies observed near the object edges and improve the detail level in the reconstructed 3D surfaces. Additionally, exploring the application of the proposed TD framework in other domains or extending it to handle more complex scenes with occlusions and varying lighting conditions could be valuable directions for future investigations. Exploring more advanced network models [82,83,84,85] (e.g., Attention UNet, R2U-Net, ResUNet, U${}^{2}$-Net, etc.) as alternatives to UNet for achieving even higher accuracy in shape measurement could be an exciting avenue for future research. As a preliminary step, we have conducted initial experiments with the proposed technique using the Attention UNet model, and the results have been summarized in Table 2. However, to draw definitive conclusions, a more comprehensive investigation is necessary in the future to make an accurate comparison. The preliminary findings indicate differing outcomes for the DFFS and TFFS datasets, with improved accuracy observed in the TFFS dataset, while there is a slight reduction in accuracy for the DFFS dataset.

## 5. Conclusions

In summary, this manuscript presents a novel time-distributed framework for 3D reconstruction by integrating fringe projection technique and deep learning. The proposed framework uses a single network and a time-distributed wrapper to convert fringe patterns to their corresponding numerators and denominators. Unlike previous approaches employing multi-stage or spatial networks, this framework utilizes the same network parameters to ensure consistent feature learning across time steps. It enables the learning of temporal dependencies among different phase-shifting frequencies. Quantitative evaluations and qualitative 3D reconstructions were conducted to validate the proposed technique, highlighting its potential for industrial applications and its contribution as a novel concept in scientific research.

## Figures and Tables

**Table 1:** Quantitative analysis comparing TD and spatial F2ND approaches.

Dataset	Method	Error (Lower Is Better)				Accuracy (Higher Is Better)		
		rel	rms	log	rms log	δ<1.25	$\delta <1.25^{2}$	$\delta <1.25^{3}$
DFFS	TD Layer	0.004	1.312	0.004	0.059	94.1%	96.6%	98.2%
	TD Module	0.004	1.216	0.004	0.055	94.9%	97.0%	98.4%
	Spatial F2ND	0.004	0.856	0.002	0.044	97.9%	98.7%	99.2%
TFFS	TD Layer	0.003	0.213	0.002	0.037	99.4%	99.5%	99.5%
	TD Module	0.003	0.176	0.002	0.035	96.9%	97.0%	97.2%
	Spatial F2ND	0.005	1.056	0.002	0.038	96.8%	96.9%	97.0%

**Table 2:** Initial quantitative evaluation of TD Module and spatial F2ND techniques using the internal Attention UNet network.

Dataset	Method	Error (Lower Is Better)				Accuracy (Higher Is Better)		
		rel	rms	log	rms log	δ<1.25	$\delta <1.25^{2}$	$\delta <1.25^{3}$
DFFS	Attention TD Module	0.003	1.334	0.005	0.060	93.6%	96.3%	98.1%
	Attention F2ND	0.005	1.345	0.004	0.058	94.1%	96.4%	98.0%
TFFS	Attention TD Module	0.003	0.150	0.002	0.035	97.0%	97.1%	97.3%
	Attention F2ND	0.005	0.941	0.002	0.040	96.9%	97.0%	97.1%

## Data Availability

The data presented in this study are available on request from the corresponding author.
